# “*Starting to think that way from the start”*: approaching deprescribing decision-making for people accessing palliative care - a qualitative exploration of healthcare professionals views

**DOI:** 10.1186/s12904-024-01523-2

**Published:** 2024-09-06

**Authors:** Anna Robinson-Barella, Charlotte Lucy Richardson, Zana Bayley, Andy Husband, Andy Bojke, Rona Bojke, Catherine Exley, Barbara Hanratty, Joanna Elverson, Jesse Jansen, Adam Todd

**Affiliations:** 1 School of Pharmacy, Newcastle University, King George VI Building, Newcastle upon Tyne, NE1 7RU UK; 2 Population Health Sciences Institute, Newcastle University, Newcastle upon Tyne, NE2 4BN UK; 3 Patient and Public Involvement, Newcastle University, Newcastle upon Tyne, NE1 7RU UK; 4 St. Oswald’s Hospice, Regent Avenue, Newcastle Upon Tyne, NE3 1EE UK; 5 Faculty of Health, Medicine and Life Sciences, Maastricht University, Maastricht, The Netherlands

**Keywords:** Deprescribing, Life-limiting illness, Medicines, Palliative care, Polypharmacy, Qualitative

## Abstract

**Background:**

Deprescribing has been defined as the planned process of reducing or stopping medications that may no longer be beneficial or are causing harm, with the goal of reducing medication burden while improving patient quality of life. At present, little is known about the specific challenges of decision-making to support deprescribing for patients who are accessing palliative care. By exploring the perspectives of healthcare professionals, this qualitative study aimed to address this gap, and explore the challenges of, and potential solutions to, making decisions about deprescribing in a palliative care context.

**Methods:**

Semi-structured interviews were conducted with healthcare professionals in-person or *via* video call, between August 2022 – January 2023. Perspectives on approaches to deprescribing in palliative care; when and how they might deprescribe; and the role of carers and family members within this process were discussed. Interviews were audio-recorded and transcribed verbatim. Reflexive thematic analysis enabled the development of themes. QSR NVivo (Version 12) facilitated data management. Ethical approval was obtained from the NHS Health Research Authority (ref 305394).

**Results:**

Twenty healthcare professionals were interviewed, including: medical consultants, nurses, specialist pharmacists, and general practitioners (GPs). Participants described the importance of deprescribing decision-making, and that it should be a considered, proactive, and planned process. Three themes were developed from the data, which centred on: (1) professional attitudes, competency and responsibility towards deprescribing; (2) changing the culture of deprescribing; and (3) involving the patient and family/caregivers in deprescribing decision-making.

**Conclusions:**

This study sought to explore the perspectives of healthcare professionals with responsibility for making deprescribing decisions with people accessing palliative care services. A range of healthcare professionals identified the importance of supporting decision-making in deprescribing, so it becomes a proactive process within a patient’s care journey, rather than a reactive consequence. Future work should explore how healthcare professionals, patients and their family can be supported in the shared decision-making processes of deprescribing.

**Trial registration:**

Ethical approval was obtained from the NHS Health Research Authority (ref 305394).

**Supplementary Information:**

The online version contains supplementary material available at 10.1186/s12904-024-01523-2.

## Background

Deprescribing has been defined as the planned process, under the supervision of healthcare professionals, of reducing or stopping medications that may no longer be beneficial or are causing harm, with the goal of reducing medication burden while improving patient quality of life [1–3]. On a global level, there is a growing recognition of the need for deprescribing [4], with the World Health Organisation (WHO) including it as a component of safe medication management, stating the process should be ‘as robust as that of prescribing’ [5]. In a palliative care context, where polypharmacy is common and medication burden is high [6, 7], deprescribing is an increasingly important care consideration. Indeed, there is growing evidence that deprescribing can be a safe and effective way to improve outcomes for patients using potentially inappropriate medication [8–10]. For example, studies concerning the deprescribing of anti-hypertensives, diuretics, benzodiazepines, antipsychotics and statins have demonstrated physical and cognitive benefits, and no significant harm, when these medications are reduced or stopped [11–14]. Studies have also shown that deprescribing of medications for patients with life limiting illness can improve quality of life [15], as well as reducing the risk of developing adverse drug events [16]. Whilst evidence has demonstrated that some interventions can be used to support deprescribing, practical challenges still remain; these can influence a decision to stop or reduce medication - especially in a palliative care context. For example: how to involve a multi-disciplinary team of healthcare professionals, patients and their family members when making shared decisions about possible deprescribing [3]. A recent systematic review explored these challenges further and highlighted the importance of involving all stakeholders in the deprescribing decision-making process to ensure a joint decision is made between the patient and healthcare professional [17]. Whilst several studies have focused on the broader issues of taking steps towards deprescribing in a palliative care context [18–21], at present, little is known about the specific challenges of decision-making that support deprescribing to occur. By exploring the perspectives of healthcare professionals with responsibility for prescribing medication, this qualitative study aimed to address this gap, explore the challenges of, and potential solutions to, making decisions about deprescribing in a palliative care context.

## Methods

### Recruitment and sampling

The consolidated criteria for reporting qualitative research (COREQ) checklist was followed (see Item 1, Supplementary File) [22]. Inclusion criteria comprised: healthcare professionals (such as nurses, pharmacists and medical doctors) with experience of prescribing or reviewing medication for patients in receipt of palliative care. To be included in the study, healthcare professionals had to practice in the UK. Recruitment was facilitated by (i) two hospital and two hospice research sites in the North East of England, United Kingdom (UK), (ii) professional palliative care networks of the research team and (iii) social media. All interested participants were emailed a participant information sheet and consent form detailing the purpose and aims of the research. Those who expressed an interest and gave their informed written consent, were enrolled in the study. There was no relationship established between the researcher and participants prior to study commencement or recruitment. Convenience sampling was used to recruit participants from a variety of job roles and responsibilities, as well as providing generalist or specialist palliative care. Practitioner age and time qualified were also considered within the sampling framework.

### Semi-structured interviews

In-depth, semi-structured interviews were conducted by one researcher (ZB, a female postdoctoral researcher with experience of qualitative research) between August 2022 and January 2023. Interviews were conducted remotely (*via* Zoom) or in-person (face-to-face) depending on participant preference. The semi-structured interview topic guide (see Item 2, Supplementary File for the complete interview guide) was developed based on three pilot interviews (not included in the final participant numbers) and was informed by findings from previous studies conducted by the research team [23, 24], as well as the lived-experiences of patient champions involved in this study (AB and RB). Areas explored included: participants’ perspectives on, and approaches to, deprescribing; when and how they might deprescribe; the role of carers and family members within this process; and exploration of gaps around deprescribing decision-making.

### Data analysis

All semi-structured interviews were audio-recorded to enable data analysis. The audio files were encrypted and transcribed verbatim by an external transcription company; audio files were uploaded to an encrypted, password-protected site and immediately following confirmation of accurate transcription were deleted. All interview data were anonymised at the point of transcription and all transcripts were checked for accuracy and correctness by ZB and CLR (a researcher with expertise in qualitative research). Participants did not provide comment on the transcripts, nor feedback on results.

Following reflexive thematic analysis processes, as defined by Braun and Clarke [25, 26], the principle of constant comparison guided an iterative process of data collection and analysis. Reflexive thematic analysis was performed by two researchers (CLR and AR-B, a researcher with expertise in conducting qualitative research) to analyse the interview data. Close and detailed reading of the transcripts allowed the researchers to familiarise themselves with the data. Initial descriptive codes were identified in a systematic manner across the data; these were then sorted into common coding patterns, which enabled the development of analytic themes from the data. The themes were reviewed, refined and named once coherent and distinctive. Two authors (AR-B and CLR) performed the data analysis through discussion and, if agreement was not reached, by consensus with the other members of the research team (AT, AH and CE, with expertise in palliative care and qualitative research). Post-interview field notes enhanced this reflective process. NVivo (version 12) software was used to facilitate data management. The research team were in agreement that data sufficiency and information power [27, 28] occurred after 18 semi-structured interviews and thus, study recruitment stopped following interview number 20; recurring similarity within participant responses, with no new concepts discussed, guided this decision. To ensure confidentiality when using direct participant quotes, non-identifiable pseudonyms are used throughout the research (e.g., Participant 1 and Participant 2 etc.)

### Ethical approval

This study was granted ethical approval by the UK National Health Service (NHS) Health Research Authority (reference 305394, date approved: 08.04.2022, South Birmingham REC). Research governance was followed in accordance with Newcastle University and NHS Trust research policies at study sites.

## Results

### Participant demographics

Twenty participants were recruited and interviewed for this study (participant demographics are described in Table 1). Of the twenty participants interviewed; 7 described their job role as a medical consultant working within various settings in the UK (including palliative medicine and respiratory medicine), 6 participants were nurses providing palliative and end-of-life care to patients across primary or secondary care settings, 4 were specialist pharmacists working across a range of disciplines, including frailty and heart failure, and 3 participants were general practitioners (GPs). Nineteen participants self-reported their ethnicity as White British and one participant identified as British Asian. Twelve of the 20 participants were aged between 40 and 50 years and the mean time qualified in their respective roles was 17 years (SD ± 8.5 years). Five interviews were conducted using video software (Zoom^®^, *n* = 5), while the rest were carried out in-person (*n* = 15). Interviews ranged from 27 to 62 min. There were no refusals to partake, participant dropouts or repeat interviews.

**Table 1:** Participant demographics

Interview number	Participant role	Sex	Age (years)	Self-reported Ethnicity	Time qualified (years)	Interview duration (mins)	Interview format
1	Nurse (Secondary Care)	Female	40–50	White British	24	27	In-person
2	Specialist Pharmacist (Heart Failure)	Female	40–50	White British	20	42	In-person
3	Specialist Pharmacist (Frailty)	Female	40–50	White British	16	33	In-person
4	Medical Consultant (Palliative Medicine)	Female	30–40	White British	14	33	Remote
5	Medical Consultant (Palliative Medicine)	Female	40–50	White British	14	62	In-person
6	Medical Consultant (Respiratory Medicine and General Medicine)	Male	60+	White British	40	59	In-person
7	Medical Consultant (Palliative Medicine)	Female	40–50	White British	18	59	In-person
8	General Practitioner (GP)	Female	40–50	White British	21	35	In-person
9	Medical Consultant (Palliative Medicine)	Female	40–50	White British	20	35	Remote
10	Consultant physician (Respiratory Medicine)	Female	40–50	White British	16	45	In-person
11	General Practitioner (GP)	Male	40–50	White British	6	49	In-person
12	Nurse Specialist (Palliative Care)	Female	50–60	White British	28	57	Remote
13	Specialist Pharmacist (Frailty)	Female	40–50	White British	18	47	In-person
14	General Practitioner (GP)	Female	30–40	British Asian	16	54	Remote
15	Medical Consultant (Palliative Medicine)	Female	40–50	White British	17	54	Remote
16	District Nurse	Female	50–60	White British	21	43	In-person
17	Specialist Pharmacist (Palliative Care)	Female	20–30	White British	2	44	In-person
18	Nurse (Secondary Care)	Female	40–50	White British	12	43	In-person
19	Palliative Care Community Nurse	Female	30–40	White British	2	36	In-person
20	Palliative Care Community Nurse	Female	30–40	White British	14	48	In-person

**Fig. 1:**
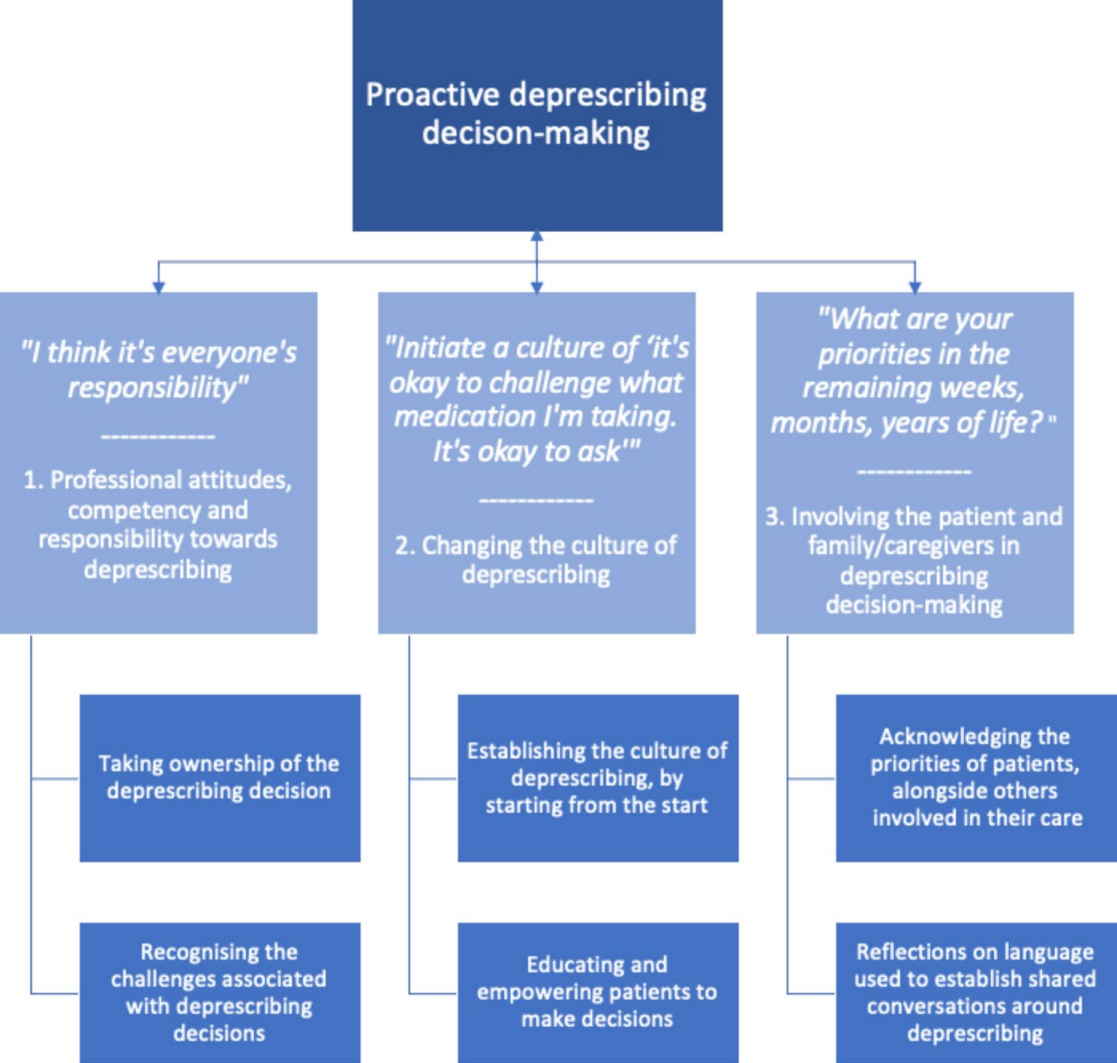
Themes developed to support the delivery of deprescribing as a proactive process, rather than a reactive consequence for patients in receipt of palliative care

From the interviews, many participants described the importance of deprescribing decision-making, and that it should be a considered, proactive, and planned process. To achieve this, three themes and subsequent sub-themes were developed from the data (Fig. 1); these focused on: (1) professional attitudes, competency and responsibility towards deprescribing; (2) changing the culture of deprescribing and (3) involving the patient and family/caregivers in deprescribing decision-making. Note the use of arrows in the figure to visually represent the interplay between the themes, and their sub-themes, as part of the bigger picture of achieving proactive deprescribing.

### Theme 1: Professional attitudes, competency and responsibility towards deprescribing

#### Taking ownership of the deprescribing decision

Participants across all healthcare professional roles shared beliefs that deprescribing was potentially a positive process; however, a number of challenges and concerns were raised, which seemed to influence whether deprescribing was undertaken, or not. The first of these related to taking ownership of the process, and ultimately, the decision-making. A number of participants described a lack of clarity around *“whose job is it to do deprescribing?”* (Participant 15). In turn, participants discussed feeling *“like I don’t want to be treading on other people’s toes”* by making decisions about stopping medicines, particularly when patients are receiving care from *“a big multidisciplinary team”* (Participant 10).

*“One of the big barriers I see is ownership… There’s a sense that “I don’t want to touch the rheumatology drugs” or “they’re under the heart failure team so I don’t want to touch any of that” … having some sense professionally*,* as a cohesive profession about who does it – I think we need to get to that place” (Participant 15*,* Medical Consultant).*

In efforts to take responsibility for deprescribing practices, participants acknowledged that certain healthcare settings may be better suited to make decisions about deprescribing. Two participants proposed making use of multidisciplinary team meetings as an approach to deprescribing decision-making in collaboration with members of the wider care team. One participant stated: *“I would always do (deprescribing) in collaboration with*,* I guess what we’d call*,* the parent team”*, indicating the connection that may be required between specialist palliative care services and other clinical specialities (Participant 12). While another participant highlighted *“the beauty of palliative care specialities (for having) an easily accessible multidisciplinary team with a doctor*,* a specialist nurse*,* a physio*,* an OT (occupational therapist) … you can sit round the table and make those decisions and offer advice”* (Participant 13). Several participants also emphasised general practice or *“care offered by teams based in the community”* as preferable settings to make decisions for deprescribing (Participant 1). For example, one participant working within secondary care shared a preference for community-based healthcare professionals to take ownership of deprescribing processes as they can *“go into somebody’s home*,* where they (the patient) will feel more comfortable… in an environment where they feel safe so that open communication can be a bit easier”* (Participant 1). The same participant indicated this preference for community-based ownership compared with conversations occurring *“if they (the patient) were on a ward setting”* (Participant 1). This was echoed by a Nurse Specialist who suggested deprescribing decision making *“needs to be done in a calmer environment when you’ve got the time to have those discussions”* (Participant 12).

#### Recognising the challenges associated with deprescribing decisions

Participants recognised the challenges that healthcare professionals face when making decisions around deprescribing medicines for patients in receipt of palliative care. One such challenge related to healthcare professionals’ fear and uncertainty about the repercussions of stopping medications. For example, the hesitancy *“of stopping medications because it is not always clear what the outcome of that is going to be”* for their patients (Participant 2).

*“I think they’re scared of stopping an aspirin and then someone having a stroke… or stopping a NOAC (non-vitamin K oral anticoagulant) and their AF (atrial fibrillation) being uncontrolled and causing a stroke. I think they’re scared of the consequences” (Participant 13*, *Specialist Pharmacist).*

Several participants suggested that this fear may relate to a lack of formal education on deprescribing for healthcare professionals during their training years with one participant stating *“F1 (foundation year 1) doctors are scared of doing deprescribing because it feels like a big responsibility*,* and that’s scary”* (Participant 13). Other participants noted caution amongst medical healthcare professionals when it came to deprescribing, and postulated that causes of this could be due to a lack of exposure in training and the lack of normalisation of the process as part of their scope of practice. One participant stated that *“if they (medics) learnt about it as undergraduates*,* like anything*,* the more you do it*,* the easier it becomes and the more confident you feel in doing it”* (Participant 3) and another suggested *“it will take a little time to become a normal thing for prescribers to do more confidently”* (Participant 13).

### Theme 2: changing the culture of deprescribing

#### Establishing the culture of deprescribing, by starting from the start

Healthcare professionals shared views around better establishing the culture of deprescribing within clinical practice. They highlighted perspectives around the importance of *“starting to think that way from the start”* (Participant 11) at the point of prescribing a medication. Participants felt that this change to prescribing culture may also prompt patients to normalise *“medicines aren’t always needed to be a forever thing”* (Participant 14).

*“When you prescribe something… historically people are just like*,* I’ve written it up and here you go and off you go. But I think maybe just having a real explanation of it… even saying there might come a point where you might want to think about stopping it” (Participant 14*,* General Practitioner).*

A key aspect within establishing a culture of deprescribing centred on participant’s views of the ‘best times’ to have conversations around stopping or reducing medication. One participant discussed a common trend seen in their clinical experience where conversations often happen too late in a patient’s clinical trajectory. They described feeling “*like it ends up being*,* if a patient is just really unwell… it’s probably then when [deprescribing] really starts being thought about”* (Participant 14). Another participant working in palliative care, echoed these views and stated *“by the time I’ve got involved as a palliative care nurse*,* it can be too late for some of these conversations. It would be nice if somebody would at least start them earlier on”* (Participant 12). Despite recognising the importance of this, the ‘optimal’ timing of these conversations was not identified by participants; instead, they placed emphasis that having conversations around deprescribing should be guided according to a person’s individualised care needs. One proposed timeframe was at the point of “*an annual medication review for everyone in respect of what you’re diagnosing and treating”* (Participant 12) and then *“continually*,* as and when”* thereafter (Participant 11).

#### Educating and empowering patients

Alongside the need for healthcare professionals to begin establishing the culture of deprescribing, it was also recognised that patient education and empowerment to make decisions around deprescribing was vital. Participants viewed deprescribing as a process ideally underpinned by shared decision-making, which should be *“collaborative… we should say (to the patient) “look*,* in a medical opinion*,* we could do this*,* but what do you think?” I think should be a proper open discussion”* (Participant 3). In order to achieve this collaboration, participants recognised the need to *“give patients information in a way that they a) want and b) understand”* so that they feel valued and *“feel a true part of the conversation”* (Participant 13). One palliative medicine consultant acknowledged the importance of patient empowerment when it came to changing the culture around deprescribing; they viewed medicines education and psychological support as vital tools to achieve supportive deprescribing and understood the worry that may accompany people having to think - and make decisions - about potentially stopping medications.

*“It’s probably the first time they’ve been asked*,* “What does this drug really mean to you? Would it worry you if you had to stop it? What do you think it’s doing for you?” Those are quite big conversations.” (Participant 15*,* Medical Consultant)*.

### Theme 3: involving patients (and others) in deprescribing decision-making

#### Acknowledging the priorities of patients, alongside others involved in their care

Healthcare professionals recognised the need to involve patients closely in conversations and decisions around deprescribing, particularly taking into consideration their priorities when it comes to medication. A number of participants reflected on the importance of individualisation and tailoring deprescribing conversations to each specific person, rather than adopting a ‘one size fits all’ approach. One participant reflected on focusing on an individual’s preferences about taking medications, recognising that there may be discrepancies between their own clinical judgement about the necessity of a medication and the desire of a person to take it; they felt it important to voice both sides of the conversation and help the patient make an informed choice about deprescribing. From this, the importance of establishing the priorities of the patient was considered essential in deprescribing decision-making.

*“Focusing on what we know what is important for that person… if somebody turned around to me and said*,* “I’m terrified of dying of a stroke*,* is it alright if I keep taking the statins?” the answer would be “absolutely yes”*,* and saying*,* “Well*,* what are your priorities in your remaining weeks*,* months*,* years of life*,* and what would you like us to focus on as healthcare professionals?” (Participant 4*,* Medical Consultant).*

Taking time to understand what influences a patient to take their medications was recognised by participants. It appeared important to appreciate that some patients may not want to make any changes with their medication at the point of an initial deprescribing conversation taking place; it was recognised that this may change over time, with fluctuations in their symptoms, disease trajectory, prognosis or even shifts in a person’s preferences or priorities around their care.

Whilst it was important to have a patient voice present in those discussions, healthcare professionals also recognised the importance of acknowledging priorities of others involved in their care, such as family, friends and caregivers. Healthcare professionals acknowledged the day-to-day input that family and carers have with their loved one’s medication, and reported it was vital to have their input into overall deprescribing decisions. One participant discussed the importance of *“pre-planning”* discussions about deprescribing with relatives, and having these conversations at an early enough stage *“when there’s less tightened emotions to give them (relative) a chance to think and input into decisions about medicines*,* at a pace that is suitable for all parties”* (Participant 3).

Participants reflected on the challenges that can sometimes present when making decisions about deprescribing if medication priorities differ between the patient and family member(s). One participant shared an example *“where the patient wants to carry on with some medication but the family thought “I don’t think you need that – it’s making you too drowsy””* (Participant 14). They went on to further postulate the implications that continuation of a medication may have for the carer *“or family member who feels like “well because you’re more drowsy*,* I’ve got to come and help you with this and that””* (Participant 14). Another participant discussed the commonality of conflicts between patients and family members when it concerns the trajectory of life-limiting illnesses. Specifically, if a carer or relative *“hasn’t accepted that the person is dying*,* and me coming in and talking about stopping all these meds they’ve been on for years – that’s often the thing that really brings it home to them”* (Participant 8).

#### Reflections on language used to establish shared conversations around deprescribing

Another participant reflected on the influence that language can have when establishing and shaping shared conversations with patients around deprescribing. They reflected on common phrasing used when prescribing and commencing new medicines, believing that often *“the message we give as professionals is “once you’re on it*,* you’re on it forever”. I get why we do that for compliance*,* concordance … but maybe we need a bit of “this will be reviewed annually and we might change it” phrasing built in”* (Participant 15). In doing so, it was deemed helpful to establish and share expectations between the prescriber and the patient relating to the intention and duration of each medication, whilst also involving patient perspectives in the ongoing review process.

Another participant shared an example where language used when starting a medication was a barrier to deprescribing in a specialist palliative care setting. They reflected on how often it is communicated by prescribers: *”never stop this medicine” or “this medicine is so important” that (when discussing deprescribing with one patient) … he just kind of thought as soon as he stopped it*,* he would die”* (Participant 17). Reflections in language were also noted by other participants, specifically when describing successful deprescribing episodes as consultations that are framed in a way to minimise (or avoid) patient fear and/or concern. They recommended using shared language that aligned with *“trialling without”* a particular medication, as opposed to *“stopping it”* (Participant 5), so that the patient felt they could have input as an equal partner in the deprescribing decision-making process.

*“It’s the framing of how you’re doing it – “we’ll put this to one side. I’m not stopping it*,* we’ll put it to one side. If you feel any different*,* you can put the medicine straight back up” (Participant 5*,* Medical Consultant).*

Another participant picked up on the influence of language when discussing deprescribing medication for patients with life-limiting illnesses. In particular, the importance of gently introducing deprescribing concepts that *“explain we’re not giving up on them”* and *“emphasising that we’re on a journey together and they’re not being left high and dry”* (Participant 9).

A further participant reflected on the importance of language and terms used to establish a relationship of trust between the healthcare professional and the patient, prior to making a deprescribing decision. For example, to help establish such a relationship they flatten the hierarchy between the patient and themselves, in a bid to “*feel that they (patients) can ask things to me that maybe they otherwise won’t”* (Participant 10). In doing so, the participant felt the dynamic of the consultation was one of shared decision-making, rather than a *“traditional consultation”* with a paternalistic approach. Other participants alluded to the power and value of forming relationships between the patient and the prescriber where *“trust needs to be at the centre … if they don’t know they can trust you*,* they might not be comfortable (with deprescribing decisions) … until you’ve built up that rapport”* (Participant 1).

## Discussion

By exploring the perspectives of healthcare professionals, this study builds on previous evidence by exploring the challenges of, and potential solutions to, making decisions about deprescribing in a palliative care context. This study collated the perspectives from a range of healthcare professional groups working with responsibility for prescribing medication in a palliative care context – a specialist patient cohort, which has previously been under-reported in the deprescribing literature, as well as healthcare research more broadly. A consistent finding across all interviews was that the decision-making to underpin deprescribing approaches should be considered, planned, and done proactively as part of ongoing clinical care, rather than as a separate reactive process as a consequence of a patient’s illness or development of an adverse drug reaction. This was a unique finding from this work, which encompassed elements of professional responsibility and attitudes, alongside a need to change the culture in which prescribing and deprescribing are recognised as similar entities when it comes to safe and effective medicines use. Another unique learning point centres on the use of shared language within deprescribing conversations; framing decision-making consultations with terminology that reflects shared, equal conversations may prove more inclusive and balanced. As well, patient empowerment and person-centred approaches were deemed essential components of deprescribing decision-making; whereby the priorities of patients and others (such as family members or carers) are recognised and included as part of the deprescribing process.

Echoing previous studies in the wider deprescribing literature [29–31], healthcare professionals interviewed as part of this study acknowledged the value of forming and establishing trust with patients prior to deprescribing conversations taking place. Indeed, given the emotions that accompany a life-limiting illness, once a positive patient-healthcare professional relationship was formed, participants perceived it was easier to broach the subject of deprescribing. It is not clear from this research if patients perceive all healthcare professionals as the same, but findings in the wider literature suggest that older adults have greater trust in certain healthcare professionals, such as medical doctors, compared to others, like pharmacists [30, 32–34]. Further building on elements of trust within deprescribing practices, participants discussed the underpinning uncertainty or fear that is experienced from a healthcare professional standpoint when a decision is made whether to deprescribe. Specifically, participants reported a lack of education built within their professional undergraduate and/or postgraduate training about how to approach deprescribing. In keeping with this study, these findings have been reported by prescribers working in a range of healthcare specialities including mental health [35], geriatric medicine [36–38] and wider primary care services [39, 40]. In recognition of the inconsistent and non-standardised implementation of deprescribing within undergraduate education, a curricular framework for approaches to deprescribing has been proposed [41]. In this, recommendations were made aiming to improve the previously reported low prescriber self-efficacy and self-confidence when it comes to deprescribing [42–47] – something which future research may seek to evaluate, specifically within specialist patient cohorts like people accessing palliative care services.

Findings in this study echoed the value of building and maintaining interdisciplinary relationships between healthcare professional groups when approaching deprescribing as part of a multi-disciplinary team [38, 48, 49]. As well as this, the significance of shared collaborative conversations between clinicians, patients, and family members or carers was also echoed [17]. Much focus within previous studies has rightly placed patient preferences of deprescribing at the centre [50–52], however, future research may wish to further explore the dynamics and interplay between the preferences of others involved in a person’s care. Considering the role that carers and family members play in supporting people in receipt of palliative care [53–56], a greater understanding of shared patient-relative-clinician triad discussions about deprescribing would be useful to explore.

Another important study finding was the importance of the language used to have shared deprescribing conversations and the language used by healthcare professionals. Studies in wider healthcare literature have previously alluded to the complexity of conversations about end-of-life care and life-limiting illness [57], as well as the challenges of initiating discussions about medicines during periods of clinical deterioration or changes in disease trajectory [58]. Findings from this work show the importance of aligning language with shared expectations and shared decisions between the prescriber and the patient, specifically relating to the intended duration and rationale for each prescribed medication. A recent study by Green et al. [59] recommended suggestions for patient-preferred language that clinicians could use when communicating deprescribing decisions amongst older adults; findings from this and other studies [60, 61] could be transferable to this work, in particular recognition of framing deprescribing decisions around an individual’s priorities and goals [59].

Whilst we believe our results are robust and have important implications for the way in which healthcare professionals approach deprescribing decision-making, we do acknowledge that the majority of our sample was limited to practice within the North East of England thus, the experience of our participants was that of, predominantly, White British females. Including the voices of healthcare professionals from other ethnic groups may have brought previously unheard considerations to the forefront, which may impact or influence deprescribing processes in minoritised groups – given the growing diversity of the patient population within the UK, this warrants further investigation in a bid to ensure cultural competence is embedded within deprescribing decision-making.

There remains a need to develop interventions to promote deprescribing decision-making for patients accessing palliative care services. In particular, there are still gaps in knowledge concerning the needs and challenges of patients and their family members or carers in this context, especially when it comes to the prescribing and deprescribing of medications as an overall component of care. Future research approaches should seek to further explore the steps within shared decision-making of deprescribing to understand how future interventions could best support this process. To achieve this, co-design methodology could be used to collectively combine the views of all people involved in the deprescribing process, including people with lived experience of receiving palliative care, their family members or carers, as well as healthcare professionals. By exploring the perspectives of healthcare professionals with responsibility for prescribing medication, this qualitative study addressed the first steps of this process and could be used to support future co-design work in this area.

## Conclusions

This study sought to further explore the perspectives of healthcare professionals with responsibility for making deprescribing decisions with people accessing palliative care services. A diverse range of healthcare professionals identified the importance of supporting deprescribing decision-making so it becomes a proactive process, using shared language, rather than a reactive consequence, within a person’s care journey. The identified themes included professional attitudes, competency and responsibility towards deprescribing decision-making; changing the culture of deprescribing; and, the importance of involving patients and their family/caregivers in deprescribing decision-making. Future work should explore how healthcare professionals, patients and their family can best be supported in the shared decision-making processes of deprescribing.

## Electronic supplementary material

Below is the link to the electronic supplementary material.

Supplementary Material 1

## Data Availability

Data is provided within the manuscript or supplementary information files.
