# Supplementary material for: “Starting to think that way from the start”: approaching deprescribing decision-making for people accessing palliative care - a qualitative exploration of healthcare professionals views
Source: BMC Palliat Care. 2024 Sep 6;23:221. doi: 10.1186/s12904-024-01523-2 (PMC11378434; doi:10.1186/s12904-024-01523-2)
Supplement: Supplementary file 1 — Supplementary Material 1 [file 12904_2024_1523_MOESM1_ESM.docx]

**Item 1:**

**COnsolidated criteria for REporting Qualitative studies (COREQ): 32-item checklist.**

| **Number** | **Item** | **Guide questions / description** | **Reported on manuscript page** |
| --- | --- | --- | --- |
| **Domain 1: research team and reflexivity** | | | |
| **Personal characteristics** | | | |
| 1 | Interviewer | Which author(s) conducted the interviews? | 5 |
| 2 | Credentials | What were the researcher’s credentials? *E.g., PhD, MD* | 5 |
| 3 | Occupation | What was their occupation at the time of the study? | 5 |
| 4 | Gender | Was the researcher male or female? | 5 |
| 5 | Experience and training | What experience or training did the researcher have? | 5 |
| **Relationship with participants** | | | |
| 6 | Relationship established | Was a relationship established prior to study commencement? | 5 |
| 7 | Participant knowledge of interviewer | What did the participants know about the researcher?  *E.g., reason for doing the research* | 5 |
| 8 | Interviewer characteristics | What characteristics were reported about the interviewer?  *E.g., bias, assumptions, reasons and interests in the research topic* | 5 |
| **Domain 2: study design** | | | |
| **Theoretical framework** | | | |
| 9 | Methodological orientation and theory | What methodological orientation was stated to underpin the study?  *E.g., grounded theory, ethnography, discourse analysis* | N/A |
| **Participant selection** | | | |
| 10 | Sampling | How were participants selected? *E.g., purposive, convenience, consecutive* | 5 |
| 11 | Method of approach | How were participants approached? *E.g., face-to-face, telephone, email* | 5 |
| 12 | Sample size | How many participants were in the study? | 7, Table 1 |
| 13 | Non-participation | How many people refused to participate or dropped out (with reasons)? | 7 |
| **Setting** | | | |
| 14 | Setting of data collection | How was the data collected? *E.g., home, clinic, workplace* | 5, 7, Table 1 |
| 15 | Presence of non-participants | Was anyone else present besides the participant and researcher? | N/A |
| 16 | Description of sample | What are the important characteristics of the sample? *E.g., demographic data* | 7, Table 1 |
| **Data collection** | | | |
| 17 | Interview guide | Were questions and prompts provided by the authors? | 5, Supplementary file |
| 18 | Repeat interviews | Were repeat interviews carried out? If yes, how many? | N/A |
| 19 | Audio/visual recording | Did the researcher use audio or visual recording to collect the data? | 6 |
| 20 | Field notes | Were field notes made during/after the interview? | 6-7 |
| 21 | Duration | What was the duration of the interviews? | Table 1 |
| 22 | Data saturation | Was data saturation discussed? | 6 |
| 23 | Transcripts returned | Were transcripts returned to participants for comment/correction? | 6 |
| **Domain 3: analysis and findings** | | | |
| **Data analysis** | | | |
| 24 | Number of data coders | How many data coders coded the data? | 6 |
| 25 | Description of the coding tree | Did authors provide a description of the coding tree? | N/A |
| 26 | Derivation of themes | Were themes identified in advance or derived from the data? | 10 |
| 27 | Software | What software, if applicable, was used to manage the data? | 6 |
| 28 | Participant checking | Did participants provide feedback on the findings? | 6 |
| **Reporting** | | | |
| 29 | Quotations presented | Were participant quotations presented to illustrate the themes / findings? Was each quotation identified? E*.g., participant number* | 12-19 |
| 30 | Data and findings consistent | Was there consistency between the data presented and the findings? | 12-19 |
| 31 | Clarity of major themes | Were major themes clearly presented in the findings? | 12-19, Figure 1 |
| 32 | Clarity of minor themes | Is there a description of diverse cases or discussion of minor themes? | 12-19, Figure 1 |

**Item 2:**

**Semi-structured interview topic guide**

The semi-structured interview questions were based around the following topic areas:

1. (Broader) Experiences of life-limiting illness in general
2. (Broad) Experiences of providing care for those with life-limiting illnesses in their place of work
3. (Narrowing) Understanding and experiences of deprescribing
4. (Focused) Examples of deprescribing within job role / within place of work
5. (Focused) Interacting with patients (and/or others) re: deprescribing
6. (Focused) Training around deprescribing – current / previous
7. (Focused) Suggestions on supporting deprescribing within life-limiting illness

The above topic guide is the full topic guide used for this study. Given the nature of semi-structured interviews, this approach enabled questions to be asked in a broad manner to start, followed by more focused questions, in a way that was adaptable for each participant (*i.e*., questions were all centred on the above topics, albeit they were worded slightly differently depending on each person’s ability to understand them, and what answers were provided in response, in order to explore fully their lived-experiences and perceptions; this is a recognised approach for qualitative research methodologies).
